# A temperature-dependent phase transformation of (*E*)-2-[(4-chloro­phen­yl)imino]­ace­naphthylen-1-one

**DOI:** 10.1107/S2056989017010659

**Published:** 2017-07-25

**Authors:** Lipiao Bao, Marilyn M. Olmstead

**Affiliations:** a School of Materials Science and Engineering, Huazhong University of Science and Technology, Wuhan 430074, Hubei, China; b Department of Chemistry, University of California, Davis, CA 95616, USA

**Keywords:** crystal structure, ordering phase transition, reversible phase transition, non-merohedral twinning, C—H⋯π inter­actions

## Abstract

The crystal structure determination based on 90 K data of the title imine ligand, C_18_H_10_ClNO, revealed non-merohedral twinning with three twin domains. In our experience, this is an indication of an ordering phase transition. Consequently, the structure was redetermined with higher temperature data, and a reversible phase transition was discovered. The higher temperature phase is indeed an ordered structure. At the higher temperature, the 4-chloro­phenyl group has rotated by *ca* 7° into a crystallographic mirror plane. Warming the crystal from 90 K to 250 K changes the space group from triclinic *P*-1, to monoclinic *P*2_1_/*m*. Diverse non-classical inter­actions are present in the crystal packing, and these are described for the phase change reported in this work. The crystal structure of the title imine ligand, measured at 100 K, has been reported on previously [Kovach *et al.* (2011 ▸). *J. Mol. Struct.* **992**, 33–38].

## Abstract 2

The crystal structure determination based on 90 K data of the title imine ligand revealed non-merohedral twinning with three twin domains. In our experience, this is an indication of an ordering phase transition. Consequently, the structure was redetermined with higher temperature data, and a reversible phase transition was discovered.

## Chemical context   

Transition metal complexes that can photochemically release carbon monoxide upon exposure to visible light have been reported recently (Chakraborty *et al.*, 2014 ▸; Stenger-Smith *et al.*, 2017 ▸). Facile release of carbon monoxide has been observed in manganese carbonyls containing ace­naphthalene derivatives (Carrington *et al.*, 2015 ▸) including the ligand MIAN {2-[(4-chloro­phen­yl)imino]­acenapthylen-1-one}, the subject of this study, shown in the Scheme. Our crystal structure determination of MIAN at 90 K agrees with the structure reported by Kovach *et al.* (2011 ▸) at 100 K. In particular, the structure occurs in the triclinic space group *P* 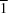 and it is found to be a twin. In the NMR study of MIAN by Kovach *et al.*, major and minor species were detected in CDCl_3_ at room temperature and a single species at 388 K in DMSO*-d*_6_. They suggested that an *E* to *Z* equilibration with the *E* form dominant takes place at the elevated temperature. The occurrence of a low-symmetry space group and twinning are indicative of a solid–solid phase change, and we were curious about the structure at higher temperatures. While a change of conformation from *E* to *Z* would be a very large solid-state change, an alternative structural change would be possible. At 250 K, a small solid-state change was indicated and the new space group is *P*2_1_/*m* (α phase). The only difference, aside from small differences in unit-cell dimensions, is a rotation of the imino­acenapthylen-1-one group into a crystallographic mirror plane. In each phase, the mol­ecule remains in the *E* conformation.
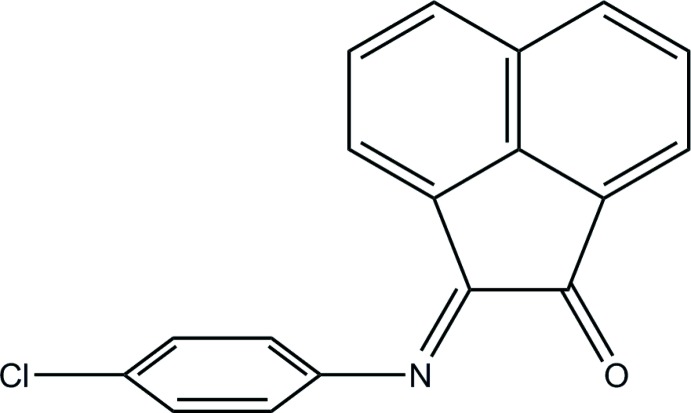


## Structural commentary   

**Figure 1:**
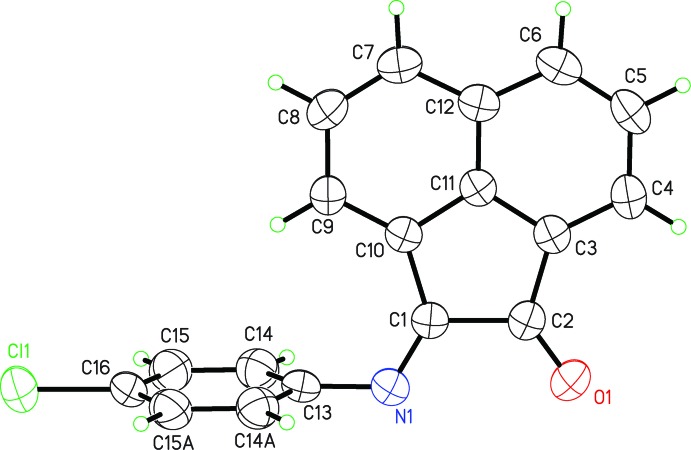
Mol­ecular structure of the title compound at 250 K (α-phase), showing 50% thermal displacement parameters and the atom-numbering scheme. Atoms C14 and C15 are related related to atoms C14*A* and C15*A*, respectively, by mirror symmetry.

**Figure 3:**
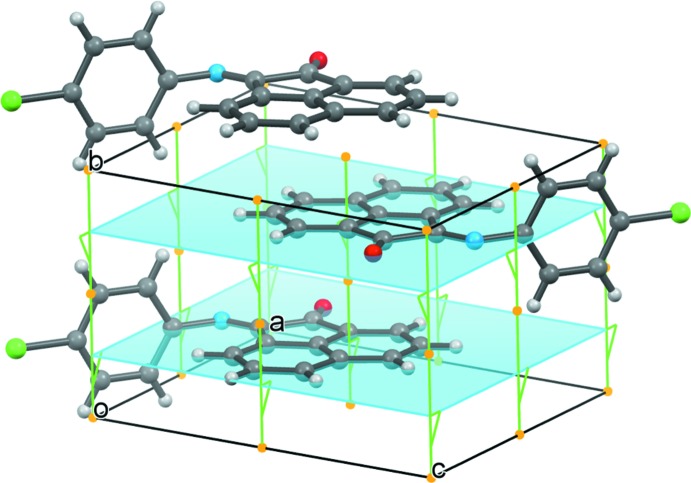
A view of the packing of the room temperature structure (α-phase). The crystallographic mirror planes are shown in blue. Orange dots indicate the crystallographic centers of inversion.

The crystal structure was initially determined at 90 K. Three twin domains were found, with relative contributions of 0.441 (2), 0.058 (3), 0.060 (3). Redetermination of the structure at higher temperatures validated our suspicion that the structure was temperature-sensitive. In order to more easily compare the low-temperature and room-temperature crystal structures, a non-standard setting for the triclinic form was selected. In this setting the shortest axis is the *b* axis. The *b* axis is then the unique axis in the monoclinic setting of *P*2_1_/m. Since minor changes in unit-cell dimensions occur, the exact temperature of the phase change was difficult to determine, but examination of the diffraction images revealed obvious twinning between 90 and 208 K, coalescence of diffraction spots occurring at 230 K, and by 250 K it was clear that the twinning had vanished and the space-group symmetry had changed. Solution of the two structures showed that the structural effect of the temperature change goes from triclinic, *P* 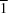 with *Z* = 2 (*Z*′ = 1) to monoclinic, *P*2_1_/*m* with *Z* = 2 (*Z*′ = 0.5). The most obvious structural change involves rotation and a change in the dihedral angle between the two mol­ecular planes that brings the acenapthyl group into the crystallographic mirror plane. At 250 K the dihedral angle is 90° while at 90 K it is 83.16 (4)°. The unit-cell volume is 2.5% larger at the higher temperature. As would be expected, thermal motion is greater at high temperature, with *U*_eq_ averaging 0.047 Å^2^ *vs* 0.017 Å^2^ at low temperature. Thermal motion in the 4-chloro­phenyl ring is slightly greater than the acenapthyl group at both temperatures, 13.5% greater in the α-phase (90 K) and 10.0% in the β-phase (250 K). Figs. 1 ▸ and 2 ▸, depict the high (α-phase) and low (β-phase) temperature structures, respectively. The similarity in the packing is evident from Figs. 3 ▸ and 4 ▸.

## Supra­molecular features   

**Figure 5:**
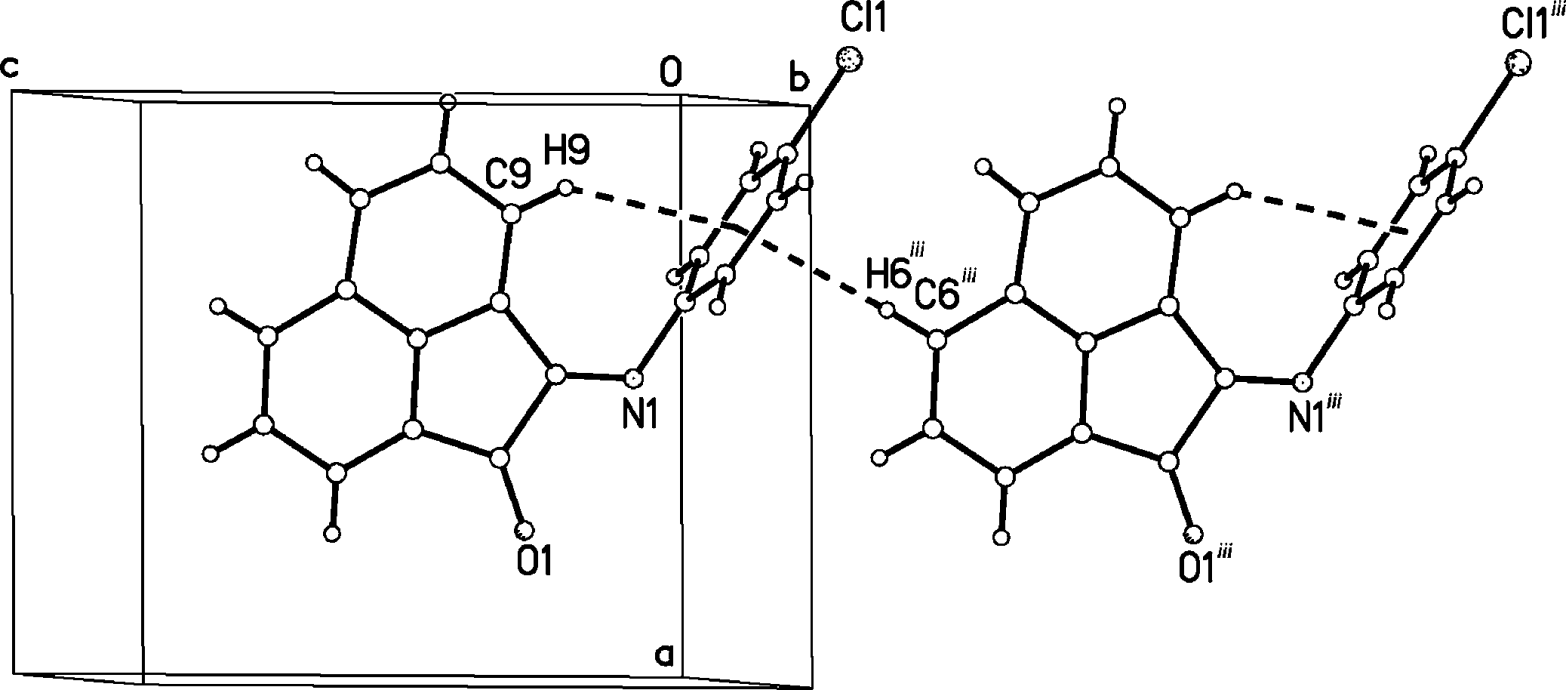
A view of the C—H⋯π inter­action linking mol­ecules together in the low temperature structure (β-phase). A similar inter­action occurs in the room-temperature structure (α-phase). Symmetry code: (iii) *x*, *y*, *z* − 1.

The two rings are perpendicular within each polymorph, likely due to a steric effect between H9, bonded to C9, and one of the *ortho* hydrogen atoms on the 4-chloro­phenyl ring (with centroid *Cg*). As a result of the perpendicular arrangement of the two ring systems, there is an intra­molecular H9⋯*Cg* distance of 2.90 Å in the 250 K structure and 2.85 Å in the 90 K structure (Tables 1 ▸ and 2 ▸). Neither structure has solvent-accessible voids. We looked for intra- and inter­molecular inter­actions that might be influential in the structural change. The only significant non-classical hydrogen bond of the C—H⋯*A* type present is found in the crystal structure of the low-temperature structure (β-phase), with a C—H⋯Cl^i^ hydrogen bond linking neighbouring mol­ecules to form chains along the *c*-axis direction (Table 2 ▸). There is, however, π–π stacking between the acenapthyl groups in each case: the inter­planar distance is 3.438 Å at 250 K and 3.409 Å at 90 K. In both phases there is a C—H⋯π inter­action on both sides of the phenyl ring, one intra­molecular and one inter­molecular (Tables 1 ▸ and 2 ▸, and Fig. 5 ▸). Temperature-driven phase changes such as this one that occur without major structural reorganization or ordering transitions have been reported in many cases: see, for example, Takahashi & Ito (2010 ▸) and Takanabe *et al.* (2017 ▸) and references therein.

## Synthesis and crystallization   

(*E*)-2-[(4-Chloro­phen­yl)imino]­ace­naphthylen-1-one (MIAN) was synthesized following a reported procedure (Kovach *et al.*, 2011 ▸). Yellow block-like crystals were obtained by layering technical grade mixed hexa­nes over a solution of the compound in CH_2_Cl_2_.

## Refinement   

Crystal data, data collection and structure refinement details are summarized in Table 3 ▸. For both polymorphs, H atoms were included in calculated positions and treated as riding: C—H = 0.94 Å in the high temperature α-phase and 0.95 Å in the low temperature β-phase, with *U*_iso_(H) = 1.2*U*_eq_(C).

## Supplementary Material

Crystal structure: contains datablock(s) alpha, beta, New_Global_Publ_Block. DOI: 10.1107/S2056989017010659/su5384sup1.cif

Structure factors: contains datablock(s) alpha. DOI: 10.1107/S2056989017010659/su5384alphasup2.hkl

Structure factors: contains datablock(s) beta. DOI: 10.1107/S2056989017010659/su5384betasup3.hkl

Supporting information file. DOI: 10.1107/S2056989017010659/su5384alphasup4.cml

Supporting information file. DOI: 10.1107/S2056989017010659/su5384betasup5.cml

CCDC references: 1563032, 1563031

Additional supporting information:  crystallographic information; 3D view; checkCIF report

## Figures and Tables

**Figure 2:**
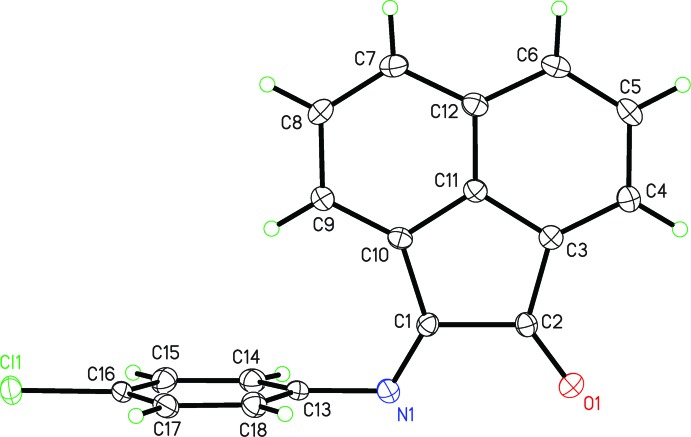
Mol­ecular structure of the title compound at 90 K (β-phase), showing 50% thermal displacement parameters and the atom-numbering scheme.

**Figure 4:**
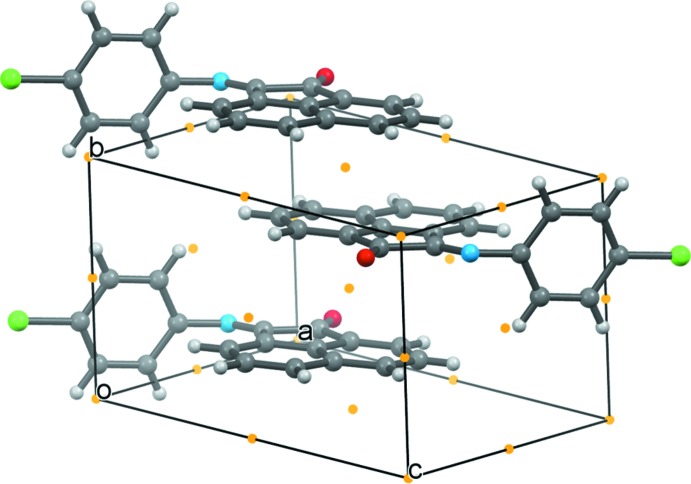
A view of the packing of the low temperature structure (β-phase). Orange dots indicate the crystallographic centers of inversion.

**Table 1:** Hydrogen-bond geometry (Å, °) for the α-phase[Chem scheme1] *Cg* is the centroid of the 4-chloro­phenyl ring (C13–C16/C14*A*/C15*A*).

*D*—H⋯*A*	*D*—H	H⋯*A*	*D*⋯*A*	*D*—H⋯*A*
C6—H6⋯*Cg* ^i^	0.94	2.86	3.803 (2)	177
C9—H9⋯*Cg*	0.94	2.88	3.668 (11)	128

Symmetry code: (i) 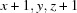.

**Table 2:** Hydrogen-bond geometry (Å, °) for the β-phase[Chem scheme1] *Cg* is the centroid of the 4-chloro­phenyl ring (C13–C18).

*D*—H⋯*A*	*D*—H	H⋯*A*	*D*⋯*A*	*D*—H⋯*A*
C7—H7⋯Cl1^i^	0.95	2.80	3.748 (2)	179
C6—H6⋯*Cg* ^ii^	0.95	2.75	3.698 (4)	177
C9—H9⋯*Cg*	0.95	2.87	3.644 (4)	142

Symmetry codes: (i) 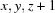; (ii) 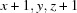.

**Table 3:** Experimental details

	α-phase	β-phase
Crystal data		
Chemical formula	C_18_H_10_ClNO	C_18_H_10_ClNO
*M*_r_	291.72	291.72
Crystal system, space group	Monoclinic, *P*2_1_/*m*	Triclinic, *P* 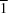
Temperature (K)	250	90
*a*, *b*, *c* (Å)	9.0447 (12), 6.8764 (9), 10.9021 (14)	9.0764 (10), 6.8187 (8), 10.7450 (12)
α, β, γ (°)	90, 92.959 (2), 90	90.880 (2), 92.780 (2), 96.259 (2)
*V* (Å^3^)	677.15 (15)	660.12 (13)
*Z*	2	2
Radiation type	Mo *K*α	Mo *K*α
μ (mm^−1^)	0.28	0.29
Crystal size (mm)	0.30 × 0.20 × 0.20	0.30 × 0.20 × 0.20

Data collection		
Diffractometer	Bruker APEXII	Bruker APEXII
Absorption correction	Multi-scan (*SADABS*; Bruker, 2014 ▸)	Multi-scan (TWINABS; Bruker, 2014 ▸)
*T*_min_, *T*_max_	0.684, 0.745	0.629, 0.746
No. of measured, independent and observed [*I* > 2σ(*I*)] reflections	5458, 1496, 1227	34083, 2949, 2726
*R*_int_	0.022	0.023
(sin θ/λ)_max_ (Å^−1^)	0.625	0.652

Refinement		
*R*[*F* ^2^ > 2σ(*F* ^2^)], *wR*(*F* ^2^), *S*	0.039, 0.097, 1.04	0.031, 0.092, 1.08
No. of reflections	1496	2949
No. of parameters	121	193
H-atom treatment	H-atom parameters constrained	H-atom parameters constrained
Δρ_max_, Δρ_min_ (e Å^−3^)	0.26, −0.38	0.33, −0.24

Computer programs: *APEX2* and *SAINT* (Bruker, 2014 ▸), *SHELXT* (Sheldrick, 2015*a* ▸), *SHELXL2016/6* (Sheldrick, 2015*b* ▸), *SHELXTL* (Sheldrick, 2008 ▸) and *Mercury* (Macrae *et al.*, 2008 ▸).

## supplementary crystallographic information

### (E)-2-[(4-Chlorophenyl)imino]acenaphthylen-1-one (alpha) Crystal data

**Table:**

C_18_H_10_ClNO	*F*(000) = 300
*M**_r_* = 291.72	*D*_x_ = 1.431 Mg m^−^^3^
Monoclinic, *P*2_1_/*m*	Mo *K*α radiation, λ = 0.71073 Å
*a* = 9.0447 (12) Å	Cell parameters from 1908 reflections
*b* = 6.8764 (9) Å	θ = 5.7–52.3°
*c* = 10.9021 (14) Å	µ = 0.28 mm^−^^1^
β = 92.959 (2)°	*T* = 250 K
*V* = 677.15 (15) Å^3^	Block, yellow
*Z* = 2	0.30 × 0.20 × 0.20 mm

### (E)-2-[(4-Chlorophenyl)imino]acenaphthylen-1-one (alpha) Data collection

**Table:**

Bruker APEXII diffractometer	1496 independent reflections
Radiation source: fine focus sealed tube	1227 reflections with *I* > 2σ(*I*)
Detector resolution: 8.3 pixels mm^-1^	*R*_int_ = 0.022
ω scans	θ_max_ = 26.4°, θ_min_ = 2.3°
Absorption correction: multi-scan (SADABS; Bruker, 2014)	*h* = −10→11
*T*_min_ = 0.684, *T*_max_ = 0.745	*k* = −8→8
5458 measured reflections	*l* = −13→13

### (E)-2-[(4-Chlorophenyl)imino]acenaphthylen-1-one (alpha) Refinement

**Table:**

Refinement on *F*^2^	Primary atom site location: dual
Least-squares matrix: full	Secondary atom site location: difference Fourier map
*R*[*F*^2^ > 2σ(*F*^2^)] = 0.039	Hydrogen site location: inferred from neighbouring sites
*wR*(*F*^2^) = 0.097	H-atom parameters constrained
*S* = 1.04	*w* = 1/[σ^2^(*F*_o_^2^) + (0.037*P*)^2^ + 0.2756*P*] where *P* = (*F*_o_^2^ + 2*F*_c_^2^)/3
1496 reflections	(Δ/σ)_max_ < 0.001
121 parameters	Δρ_max_ = 0.26 e Å^−^^3^
0 restraints	Δρ_min_ = −0.38 e Å^−^^3^

### (E)-2-[(4-Chlorophenyl)imino]acenaphthylen-1-one (alpha) Special details

**Table:**

Geometry. All esds (except the esd in the dihedral angle between two l.s. planes) are estimated using the full covariance matrix. The cell esds are taken into account individually in the estimation of esds in distances, angles and torsion angles; correlations between esds in cell parameters are only used when they are defined by crystal symmetry. An approximate (isotropic) treatment of cell esds is used for estimating esds involving l.s. planes.

### (E)-2-[(4-Chlorophenyl)imino]acenaphthylen-1-one (alpha) Fractional atomic coordinates and isotropic or equivalent isotropic displacement parameters (Å^2^)

**Table:**

	*x*	*y*	*z*	*U*_iso_*/*U*_eq_	
Cl1	−0.05474 (8)	0.250000	−0.19593 (6)	0.0739 (3)	
O1	0.75377 (18)	0.250000	0.29014 (16)	0.0547 (5)	
N1	0.4932 (2)	0.250000	0.12642 (17)	0.0442 (5)	
C1	0.4858 (2)	0.250000	0.2426 (2)	0.0366 (5)	
C2	0.6292 (2)	0.250000	0.3259 (2)	0.0395 (5)	
C3	0.5810 (2)	0.250000	0.4537 (2)	0.0366 (5)	
C4	0.6571 (3)	0.250000	0.5661 (2)	0.0440 (6)	
H4	0.761142	0.250000	0.571881	0.053*	
C5	0.5750 (3)	0.250000	0.6722 (2)	0.0475 (6)	
H5	0.626114	0.250000	0.749460	0.057*	
C6	0.4230 (3)	0.250000	0.6670 (2)	0.0473 (6)	
H6	0.372512	0.250000	0.740235	0.057*	
C7	0.1855 (3)	0.250000	0.5310 (2)	0.0564 (7)	
H7	0.124153	0.250000	0.598029	0.068*	
C8	0.1232 (3)	0.250000	0.4135 (2)	0.0584 (7)	
H8	0.019501	0.250000	0.402169	0.070*	
C9	0.2096 (2)	0.250000	0.3087 (2)	0.0455 (6)	
H9	0.164153	0.250000	0.229206	0.055*	
C10	0.3610 (2)	0.250000	0.32570 (19)	0.0361 (5)	
C11	0.4247 (2)	0.250000	0.44731 (19)	0.0347 (5)	
C12	0.3414 (3)	0.250000	0.5522 (2)	0.0419 (5)	
C13	0.3601 (2)	0.250000	0.05083 (19)	0.0398 (5)	
C14	0.29812 (19)	0.0762 (3)	0.00977 (15)	0.0479 (4)	
H14	0.342774	−0.042208	0.033729	0.058*	
C15	0.17066 (19)	0.0757 (3)	−0.06642 (15)	0.0514 (5)	
H15	0.127910	−0.042285	−0.093524	0.062*	
C16	0.1076 (3)	0.250000	−0.1018 (2)	0.0471 (6)	

### (E)-2-[(4-Chlorophenyl)imino]acenaphthylen-1-one (alpha) Atomic displacement parameters (Å^2^)

**Table:**

	*U*^11^	*U*^22^	*U*^33^	*U*^12^	*U*^13^	*U*^23^
Cl1	0.0487 (4)	0.1311 (8)	0.0411 (4)	0.000	−0.0069 (3)	0.000
O1	0.0345 (9)	0.0793 (13)	0.0506 (10)	0.000	0.0053 (7)	0.000
N1	0.0405 (10)	0.0580 (13)	0.0342 (10)	0.000	0.0025 (8)	0.000
C1	0.0366 (11)	0.0376 (12)	0.0357 (12)	0.000	0.0026 (9)	0.000
C2	0.0357 (12)	0.0408 (13)	0.0420 (12)	0.000	0.0007 (9)	0.000
C3	0.0395 (12)	0.0338 (11)	0.0364 (11)	0.000	−0.0005 (9)	0.000
C4	0.0430 (13)	0.0447 (14)	0.0433 (13)	0.000	−0.0084 (10)	0.000
C5	0.0613 (16)	0.0465 (14)	0.0335 (12)	0.000	−0.0088 (11)	0.000
C6	0.0611 (16)	0.0473 (14)	0.0337 (12)	0.000	0.0060 (11)	0.000
C7	0.0450 (14)	0.081 (2)	0.0442 (14)	0.000	0.0130 (11)	0.000
C8	0.0350 (12)	0.089 (2)	0.0517 (15)	0.000	0.0075 (11)	0.000
C9	0.0361 (12)	0.0619 (16)	0.0383 (12)	0.000	−0.0010 (9)	0.000
C10	0.0369 (11)	0.0374 (12)	0.0340 (11)	0.000	0.0025 (9)	0.000
C11	0.0379 (11)	0.0312 (11)	0.0350 (11)	0.000	0.0005 (9)	0.000
C12	0.0458 (13)	0.0424 (13)	0.0377 (12)	0.000	0.0056 (10)	0.000
C13	0.0396 (12)	0.0523 (14)	0.0280 (10)	0.000	0.0072 (9)	0.000
C14	0.0494 (9)	0.0480 (10)	0.0465 (9)	0.0010 (8)	0.0034 (7)	0.0022 (8)
C15	0.0499 (10)	0.0593 (12)	0.0452 (9)	−0.0073 (9)	0.0038 (8)	−0.0108 (9)
C16	0.0401 (12)	0.0743 (18)	0.0269 (11)	0.000	0.0028 (9)	0.000

### (E)-2-[(4-Chlorophenyl)imino]acenaphthylen-1-one (alpha) Geometric parameters (Å, º)

**Table:**

Cl1—C16	1.747 (2)	C7—C8	1.372 (4)
O1—C2	1.211 (3)	C7—C12	1.417 (3)
N1—C1	1.272 (3)	C7—H7	0.9400
N1—C13	1.423 (3)	C8—C9	1.417 (3)
C1—C10	1.484 (3)	C8—H8	0.9400
C1—C2	1.545 (3)	C9—C10	1.372 (3)
C2—C3	1.481 (3)	C9—H9	0.9400
C3—C4	1.373 (3)	C10—C11	1.418 (3)
C3—C11	1.413 (3)	C11—C12	1.402 (3)
C4—C5	1.406 (3)	C13—C14	1.385 (2)
C4—H4	0.9400	C13—C14^i^	1.385 (2)
C5—C6	1.373 (4)	C14—C15	1.386 (2)
C5—H5	0.9400	C14—H14	0.9400
C6—C12	1.420 (3)	C15—C16	1.374 (2)
C6—H6	0.9400	C15—H15	0.9400
			
C1—N1—C13	119.39 (19)	C10—C9—C8	118.6 (2)
N1—C1—C10	133.5 (2)	C10—C9—H9	120.7
N1—C1—C2	120.02 (19)	C8—C9—H9	120.7
C10—C1—C2	106.45 (17)	C9—C10—C11	118.7 (2)
O1—C2—C3	128.8 (2)	C9—C10—C1	134.7 (2)
O1—C2—C1	125.3 (2)	C11—C10—C1	106.63 (18)
C3—C2—C1	105.91 (18)	C12—C11—C3	122.6 (2)
C4—C3—C11	119.9 (2)	C12—C11—C10	123.6 (2)
C4—C3—C2	132.9 (2)	C3—C11—C10	113.79 (19)
C11—C3—C2	107.22 (19)	C11—C12—C7	116.0 (2)
C3—C4—C5	118.2 (2)	C11—C12—C6	116.3 (2)
C3—C4—H4	120.9	C7—C12—C6	127.7 (2)
C5—C4—H4	120.9	C14—C13—C14^i^	119.3 (2)
C6—C5—C4	122.4 (2)	C14—C13—N1	120.24 (11)
C6—C5—H5	118.8	C14^i^—C13—N1	120.24 (11)
C4—C5—H5	118.8	C13—C14—C15	120.43 (18)
C5—C6—C12	120.7 (2)	C13—C14—H14	119.8
C5—C6—H6	119.7	C15—C14—H14	119.8
C12—C6—H6	119.7	C16—C15—C14	119.13 (18)
C8—C7—C12	120.6 (2)	C16—C15—H15	120.4
C8—C7—H7	119.7	C14—C15—H15	120.4
C12—C7—H7	119.7	C15^i^—C16—C15	121.4 (2)
C7—C8—C9	122.4 (2)	C15^i^—C16—Cl1	119.28 (11)
C7—C8—H8	118.8	C15—C16—Cl1	119.28 (11)
C9—C8—H8	118.8		
			
C13—N1—C1—C10	0.000 (1)	C2—C3—C11—C12	180.000 (1)
C13—N1—C1—C2	180.000 (1)	C4—C3—C11—C10	180.000 (1)
N1—C1—C2—O1	0.000 (1)	C2—C3—C11—C10	0.000 (1)
C10—C1—C2—O1	180.000 (1)	C9—C10—C11—C12	0.000 (1)
N1—C1—C2—C3	180.000 (1)	C1—C10—C11—C12	180.000 (1)
C10—C1—C2—C3	0.000 (1)	C9—C10—C11—C3	180.000 (1)
O1—C2—C3—C4	0.000 (1)	C1—C10—C11—C3	0.000 (1)
C1—C2—C3—C4	180.000 (1)	C3—C11—C12—C7	180.000 (1)
O1—C2—C3—C11	180.000 (1)	C10—C11—C12—C7	0.000 (1)
C1—C2—C3—C11	0.000 (1)	C3—C11—C12—C6	0.000 (1)
C11—C3—C4—C5	0.000 (1)	C10—C11—C12—C6	180.000 (1)
C2—C3—C4—C5	180.000 (1)	C8—C7—C12—C11	0.000 (1)
C3—C4—C5—C6	0.000 (1)	C8—C7—C12—C6	180.000 (1)
C4—C5—C6—C12	0.000 (1)	C5—C6—C12—C11	0.000 (1)
C12—C7—C8—C9	0.000 (1)	C5—C6—C12—C7	180.000 (1)
C7—C8—C9—C10	0.000 (1)	C1—N1—C13—C14	−92.57 (18)
C8—C9—C10—C11	0.000 (1)	C1—N1—C13—C14^i^	92.57 (18)
C8—C9—C10—C1	180.000 (1)	C14^i^—C13—C14—C15	−3.4 (3)
N1—C1—C10—C9	0.000 (1)	N1—C13—C14—C15	−178.35 (17)
C2—C1—C10—C9	180.000 (1)	C13—C14—C15—C16	0.7 (3)
N1—C1—C10—C11	180.000 (1)	C14—C15—C16—C15^i^	2.0 (3)
C2—C1—C10—C11	0.000 (1)	C14—C15—C16—Cl1	−179.01 (14)
C4—C3—C11—C12	0.000 (1)		

Symmetry code: (i) *x*, −*y*+1/2, *z*.

### (E)-2-[(4-Chlorophenyl)imino]acenaphthylen-1-one (alpha) Hydrogen-bond geometry (Å, º)

Cg is the centroid of the 4-chlorophenyl ring (C13–C16/C14*A*/C15*A*).

**Table:**

*D*—H···*A*	*D*—H	H···*A*	*D*···*A*	*D*—H···*A*
C6—H6···*Cg*^ii^	0.94	2.86	3.803 (2)	177
C9—H9···*Cg*	0.94	2.88	3.668 (11)	128

Symmetry code: (ii) *x*+1, *y*, *z*+1.

### (E)-2-[(4-Chlorophenyl)imino]acenaphthylen-1-one (beta) Crystal data

**Table:**

C_18_H_10_ClNO	*Z* = 2
*M**_r_* = 291.72	*F*(000) = 300
Triclinic, *P*1	*D*_x_ = 1.468 Mg m^−^^3^
*a* = 9.0764 (10) Å	Mo *K*α radiation, λ = 0.71073 Å
*b* = 6.8187 (8) Å	Cell parameters from 9928 reflections
*c* = 10.7450 (12) Å	θ = 4.5–55.2°
α = 90.880 (2)°	µ = 0.29 mm^−^^1^
β = 92.780 (2)°	*T* = 90 K
γ = 96.259 (2)°	Block, yellow
*V* = 660.12 (13) Å^3^	0.30 × 0.20 × 0.20 mm

### (E)-2-[(4-Chlorophenyl)imino]acenaphthylen-1-one (beta) Data collection

**Table:**

Bruker APEXII diffractometer	2949 independent reflections
Radiation source: fine focus sealed tube	2726 reflections with *I* > 2σ(*I*)
Detector resolution: 8.3 pixels mm^-1^	*R*_int_ = 0.023
ω scans	θ_max_ = 27.6°, θ_min_ = 1.9°
Absorption correction: multi-scan (TWINABS; Bruker, 2014)	*h* = −11→11
*T*_min_ = 0.629, *T*_max_ = 0.746	*k* = −8→8
34083 measured reflections	*l* = −13→13

### (E)-2-[(4-Chlorophenyl)imino]acenaphthylen-1-one (beta) Refinement

**Table:**

Refinement on *F*^2^	Primary atom site location: dual
Least-squares matrix: full	Secondary atom site location: difference Fourier map
*R*[*F*^2^ > 2σ(*F*^2^)] = 0.031	Hydrogen site location: inferred from neighbouring sites
*wR*(*F*^2^) = 0.092	H-atom parameters constrained
*S* = 1.08	*w* = 1/[σ^2^(*F*_o_^2^) + (0.0535*P*)^2^ + 0.1518*P*] where *P* = (*F*_o_^2^ + 2*F*_c_^2^)/3
2949 reflections	(Δ/σ)_max_ < 0.001
193 parameters	Δρ_max_ = 0.33 e Å^−^^3^
0 restraints	Δρ_min_ = −0.24 e Å^−^^3^

### (E)-2-[(4-Chlorophenyl)imino]acenaphthylen-1-one (beta) Special details

**Table:**

Geometry. All esds (except the esd in the dihedral angle between two l.s. planes) are estimated using the full covariance matrix. The cell esds are taken into account individually in the estimation of esds in distances, angles and torsion angles; correlations between esds in cell parameters are only used when they are defined by crystal symmetry. An approximate (isotropic) treatment of cell esds is used for estimating esds involving l.s. planes.
Refinement. Refined as a 4-component twin.

### (E)-2-[(4-Chlorophenyl)imino]acenaphthylen-1-one (beta) Fractional atomic coordinates and isotropic or equivalent isotropic displacement parameters (Å^2^)

**Table:**

	*x*	*y*	*z*	*U*_iso_*/*U*_eq_	
Cl1	−0.06705 (4)	0.27235 (6)	−0.20019 (3)	0.02295 (12)	
O1	0.74526 (11)	0.24307 (17)	0.28273 (9)	0.0189 (2)	
N1	0.48366 (13)	0.23262 (19)	0.11662 (11)	0.0160 (3)	
C1	0.47710 (15)	0.2430 (2)	0.23456 (13)	0.0132 (3)	
C2	0.62087 (15)	0.2429 (2)	0.31934 (13)	0.0138 (3)	
C3	0.57248 (15)	0.2463 (2)	0.44935 (13)	0.0136 (3)	
C4	0.64841 (16)	0.2421 (2)	0.56328 (13)	0.0160 (3)	
H4	0.752714	0.237152	0.568827	0.019*	
C5	0.56597 (16)	0.2455 (2)	0.67222 (13)	0.0172 (3)	
H5	0.616929	0.242653	0.751402	0.021*	
C6	0.41399 (16)	0.2527 (2)	0.66699 (13)	0.0171 (3)	
H6	0.363047	0.256111	0.742037	0.021*	
C7	0.17740 (16)	0.2579 (2)	0.52922 (14)	0.0200 (3)	
H7	0.115871	0.260723	0.598122	0.024*	
C8	0.11510 (16)	0.2563 (3)	0.40886 (14)	0.0212 (3)	
H8	0.010792	0.257525	0.397112	0.025*	
C9	0.20184 (15)	0.2531 (2)	0.30235 (13)	0.0165 (3)	
H9	0.156281	0.252026	0.220822	0.020*	
C10	0.35329 (15)	0.2514 (2)	0.31941 (12)	0.0136 (3)	
C11	0.41694 (15)	0.2523 (2)	0.44327 (13)	0.0132 (3)	
C12	0.33329 (16)	0.2552 (2)	0.55003 (13)	0.0156 (3)	
C13	0.35083 (15)	0.2411 (2)	0.04076 (12)	0.0148 (3)	
C14	0.27646 (16)	0.0687 (2)	−0.01417 (13)	0.0173 (3)	
H14	0.313778	−0.054584	−0.000952	0.021*	
C15	0.14723 (16)	0.0784 (2)	−0.08846 (14)	0.0184 (3)	
H15	0.094740	−0.038308	−0.124808	0.022*	
C16	0.09615 (15)	0.2603 (2)	−0.10873 (12)	0.0167 (3)	
C17	0.17291 (16)	0.4341 (2)	−0.05980 (13)	0.0178 (3)	
H17	0.138749	0.558005	−0.077520	0.021*	
C18	0.30080 (16)	0.4234 (2)	0.01565 (13)	0.0170 (3)	
H18	0.354230	0.540777	0.050193	0.020*	

### (E)-2-[(4-Chlorophenyl)imino]acenaphthylen-1-one (beta) Atomic displacement parameters (Å^2^)

**Table:**

	*U*^11^	*U*^22^	*U*^33^	*U*^12^	*U*^13^	*U*^23^
Cl1	0.01595 (18)	0.0391 (2)	0.01433 (17)	0.00712 (15)	−0.00253 (11)	−0.00184 (15)
O1	0.0136 (5)	0.0254 (6)	0.0180 (5)	0.0036 (4)	0.0023 (4)	0.0018 (4)
N1	0.0151 (6)	0.0200 (6)	0.0133 (5)	0.0039 (5)	−0.0001 (4)	0.0016 (5)
C1	0.0122 (6)	0.0124 (6)	0.0152 (6)	0.0022 (5)	0.0006 (5)	0.0013 (5)
C2	0.0146 (6)	0.0129 (7)	0.0139 (6)	0.0023 (5)	−0.0007 (5)	0.0012 (5)
C3	0.0153 (7)	0.0105 (7)	0.0148 (6)	0.0011 (5)	0.0006 (5)	0.0000 (5)
C4	0.0166 (7)	0.0153 (7)	0.0159 (6)	0.0022 (5)	−0.0012 (5)	0.0007 (5)
C5	0.0232 (7)	0.0160 (7)	0.0123 (6)	0.0023 (5)	−0.0024 (5)	0.0008 (5)
C6	0.0226 (7)	0.0163 (7)	0.0125 (6)	0.0008 (5)	0.0034 (5)	0.0006 (5)
C7	0.0173 (7)	0.0261 (8)	0.0167 (7)	0.0011 (6)	0.0051 (5)	0.0005 (6)
C8	0.0124 (6)	0.0307 (9)	0.0207 (7)	0.0022 (6)	0.0026 (5)	0.0019 (6)
C9	0.0154 (7)	0.0203 (7)	0.0136 (6)	0.0011 (5)	−0.0002 (5)	0.0009 (5)
C10	0.0159 (7)	0.0122 (7)	0.0128 (6)	0.0013 (5)	0.0019 (5)	0.0011 (5)
C11	0.0151 (6)	0.0104 (6)	0.0140 (6)	0.0007 (5)	0.0006 (5)	0.0012 (5)
C12	0.0185 (7)	0.0140 (7)	0.0140 (6)	0.0007 (5)	0.0015 (5)	0.0007 (5)
C13	0.0129 (6)	0.0225 (8)	0.0098 (6)	0.0035 (5)	0.0024 (5)	0.0019 (5)
C14	0.0181 (7)	0.0189 (7)	0.0157 (7)	0.0047 (5)	0.0036 (5)	0.0014 (6)
C15	0.0157 (7)	0.0220 (8)	0.0173 (7)	0.0014 (5)	0.0020 (5)	−0.0035 (6)
C16	0.0123 (6)	0.0287 (8)	0.0097 (6)	0.0046 (6)	0.0011 (5)	0.0015 (6)
C17	0.0191 (7)	0.0211 (8)	0.0144 (6)	0.0066 (6)	0.0023 (5)	0.0024 (6)
C18	0.0180 (7)	0.0181 (7)	0.0149 (6)	0.0025 (5)	0.0006 (5)	−0.0011 (5)

### (E)-2-[(4-Chlorophenyl)imino]acenaphthylen-1-one (beta) Geometric parameters (Å, º)

**Table:**

Cl1—C16	1.7478 (14)	C7—H7	0.9500
O1—C2	1.2133 (17)	C8—C9	1.4214 (19)
N1—C1	1.2728 (18)	C8—H8	0.9500
N1—C13	1.4292 (17)	C9—C10	1.3793 (19)
C1—C10	1.4863 (18)	C9—H9	0.9500
C1—C2	1.5548 (18)	C10—C11	1.4246 (18)
C2—C3	1.4851 (19)	C11—C12	1.4070 (19)
C3—C4	1.3777 (19)	C13—C18	1.394 (2)
C3—C11	1.4151 (19)	C13—C14	1.398 (2)
C4—C5	1.421 (2)	C14—C15	1.395 (2)
C4—H4	0.9500	C14—H14	0.9500
C5—C6	1.384 (2)	C15—C16	1.387 (2)
C5—H5	0.9500	C15—H15	0.9500
C6—C12	1.4249 (19)	C16—C17	1.390 (2)
C6—H6	0.9500	C17—C18	1.393 (2)
C7—C8	1.385 (2)	C17—H17	0.9500
C7—C12	1.424 (2)	C18—H18	0.9500
			
C1—N1—C13	118.79 (12)	C9—C10—C11	118.72 (12)
N1—C1—C10	133.62 (13)	C9—C10—C1	134.58 (12)
N1—C1—C2	119.95 (12)	C11—C10—C1	106.67 (11)
C10—C1—C2	106.41 (11)	C12—C11—C3	122.84 (13)
O1—C2—C3	128.96 (13)	C12—C11—C10	123.41 (13)
O1—C2—C1	125.29 (12)	C3—C11—C10	113.74 (12)
C3—C2—C1	105.75 (11)	C11—C12—C7	116.47 (13)
C4—C3—C11	120.05 (13)	C11—C12—C6	116.28 (13)
C4—C3—C2	132.53 (13)	C7—C12—C6	127.25 (13)
C11—C3—C2	107.41 (12)	C18—C13—C14	120.11 (13)
C3—C4—C5	117.97 (13)	C18—C13—N1	119.71 (13)
C3—C4—H4	121.0	C14—C13—N1	120.07 (13)
C5—C4—H4	121.0	C15—C14—C13	119.71 (14)
C6—C5—C4	122.29 (13)	C15—C14—H14	120.1
C6—C5—H5	118.9	C13—C14—H14	120.1
C4—C5—H5	118.9	C16—C15—C14	119.29 (14)
C5—C6—C12	120.57 (13)	C16—C15—H15	120.4
C5—C6—H6	119.7	C14—C15—H15	120.4
C12—C6—H6	119.7	C15—C16—C17	121.63 (13)
C8—C7—C12	120.26 (13)	C15—C16—Cl1	119.34 (12)
C8—C7—H7	119.9	C17—C16—Cl1	119.02 (12)
C12—C7—H7	119.9	C16—C17—C18	118.81 (14)
C7—C8—C9	122.29 (13)	C16—C17—H17	120.6
C7—C8—H8	118.9	C18—C17—H17	120.6
C9—C8—H8	118.9	C17—C18—C13	120.32 (14)
C10—C9—C8	118.85 (13)	C17—C18—H18	119.8
C10—C9—H9	120.6	C13—C18—H18	119.8
C8—C9—H9	120.6		
			
C13—N1—C1—C10	4.4 (2)	C2—C3—C11—C10	0.57 (17)
C13—N1—C1—C2	−177.46 (12)	C9—C10—C11—C12	−0.1 (2)
N1—C1—C2—O1	3.7 (2)	C1—C10—C11—C12	−178.59 (13)
C10—C1—C2—O1	−177.65 (14)	C9—C10—C11—C3	178.77 (13)
N1—C1—C2—C3	−177.25 (13)	C1—C10—C11—C3	0.33 (17)
C10—C1—C2—C3	1.36 (14)	C3—C11—C12—C7	−178.99 (13)
O1—C2—C3—C4	−3.2 (3)	C10—C11—C12—C7	−0.2 (2)
C1—C2—C3—C4	177.87 (15)	C3—C11—C12—C6	0.3 (2)
O1—C2—C3—C11	177.79 (15)	C10—C11—C12—C6	179.11 (13)
C1—C2—C3—C11	−1.18 (15)	C8—C7—C12—C11	0.4 (2)
C11—C3—C4—C5	−0.4 (2)	C8—C7—C12—C6	−178.80 (16)
C2—C3—C4—C5	−179.39 (14)	C5—C6—C12—C11	−0.7 (2)
C3—C4—C5—C6	0.0 (2)	C5—C6—C12—C7	178.45 (15)
C4—C5—C6—C12	0.6 (2)	C1—N1—C13—C18	81.00 (18)
C12—C7—C8—C9	−0.3 (3)	C1—N1—C13—C14	−102.89 (16)
C7—C8—C9—C10	0.0 (2)	C18—C13—C14—C15	−3.6 (2)
C8—C9—C10—C11	0.2 (2)	N1—C13—C14—C15	−179.75 (12)
C8—C9—C10—C1	178.14 (16)	C13—C14—C15—C16	1.3 (2)
N1—C1—C10—C9	−0.8 (3)	C14—C15—C16—C17	1.9 (2)
C2—C1—C10—C9	−179.12 (16)	C14—C15—C16—Cl1	−179.20 (10)
N1—C1—C10—C11	177.30 (16)	C15—C16—C17—C18	−2.7 (2)
C2—C1—C10—C11	−1.04 (15)	Cl1—C16—C17—C18	178.37 (10)
C4—C3—C11—C12	0.3 (2)	C16—C17—C18—C13	0.3 (2)
C2—C3—C11—C12	179.49 (13)	C14—C13—C18—C17	2.8 (2)
C4—C3—C11—C10	−178.61 (13)	N1—C13—C18—C17	178.94 (12)

### (E)-2-[(4-Chlorophenyl)imino]acenaphthylen-1-one (beta) Hydrogen-bond geometry (Å, º)

Cg is the centroid of the 4-chlorophenyl ring (C13–C18).

**Table:**

*D*—H···*A*	*D*—H	H···*A*	*D*···*A*	*D*—H···*A*
C7—H7···Cl1^i^	0.95	2.80	3.748 (2)	179
C6—H6···*Cg*^ii^	0.95	2.75	3.698 (4)	177
C9—H9···*Cg*	0.95	2.87	3.644 (4)	142

Symmetry codes: (i) *x*, *y*, *z*+1; (ii) *x*+1, *y*, *z*+1.
